# Prevalence and risk factors of childhood anemia in urban and rural areas of West Java, Indonesia: a cross-sectional study

**DOI:** 10.1186/s12887-026-06859-7

**Published:** 2026-05-06

**Authors:** Rodman Tarigan Girsang, Muhammad Gilang Dwi Putra, Kusnandi Rusmil, Eddy Fadlyana, Riyadi Adrizain, Nur Melani Sari, Frizka Primadewi Fulendry, Alvira Dwilestarie Putri, Behesti Zahra Mardiah, Rona Kania Utami, Rizky Perdana Mulyadi, Arief Budiman, Dinda Tiaraningrum Nashsyah, Hadyana Sukandar

**Affiliations:** 1 Child Health Department, Faculty of Medicine, Universitas Padjadjaran, Bandung, Indonesia; 2 Mother and Child Health Department, Dr. Hasan Sadikin General Hospital, Bandung, Indonesia; 3 Public Health Department, Faculty of Medicine, Universitas Padjadjaran, Bandung, Indonesia

**Keywords:** Anemia, Children, Rural, Urban, 6–59 months

## Abstract

**Background:**

Anemia in children has become a serious global public health problem, which may lead to delayed growth and possibly have long term effects on neurodevelopmental and behavioral outcomes. Therefore, this study aimed to determine the prevalence and determinants of anemia among children in urban and rural areas of West Java, Indonesia.

**Methods:**

An observational analysis and cross-sectional study was conducted, with data was taken from secondary data of serosurvey of hand, foot, and mouth disease (HFMD) study of 560 healthy children aged 6–59 months in November 2022–January 2023 at the Garuda Primary Health Care in Bandung City as urban area and Padalarang Primary Health Care in West Bandung Region as rural area. The Chi-square test and logistic regression model were used to identify risk factors of anemia in urban and rural areas.

**Result:**

The results showed anemia was not significantly higher in urban areas (25.6%) than in rural areas (21.3%) with a p-value 0.220. In urban areas, anemia was significantly associated with children aged 6–23 months (AOR = 2.17 4; 95% CI: 1.44–3.26), stunting children (AOR = 1.71; 95% CI: 1.07–2.72) and children with parents income below regional minimum wage (AOR = 1.73 1.74; 95% CI: 1.14–2.63). In rural areas, no variables had a significant relationship with anemia.

**Conclusion:**

The current study showed that children in rural and urban areas can have anemia. Further research and evaluation are needed in the detection and monitoring of risk factors through a multisectoral approach.

## Introduction

Childhood anemia is a major global public health challenge, particularly in children aged 6–59 months, with profound health, developmental, and socioeconomic consequences [1–7]. This age group is especially vulnerable due to rapid growth, increased blood volume, and higher micronutrient requirements.[8–15] While iron deficiency is the most common cause, other contributors include infections, parasitic infestations, genetic hemoglobinopathies, and deficiencies of vitamin B12, folate, or vitamin A [16–22]. Anemia in early childhood is associated with impaired cognitive and motor development, reduced academic performance, increased susceptibility to infections, fatigue, and long-term deficits in growth and productivity [7, 20, 23]. Because of these serious outcomes, anemia reduction is a key target in the World Health Assembly’s Global Nutrition Targets, highlighting the need for evidence-based interventions to improve child health globally [8, 24].

22Globally, anemia affects approximately 39.8% of children under five, corresponding to roughly 269 million children, with the highest burden in low- and middle-income countries, especially in Africa and Asia [1–3]. Contributing factors include poor dietary diversity, limited access to healthcare, low socioeconomic status, and inadequate sanitation [1–3]. In Indonesia, anemia remains a critical public health problem. According to the 2013 Basic Health Research Survey (Riskesdas), 28% of children under five and 26% of children aged 5–14 years were anemic [3], while WHO 2019 estimates reported 38.4% of children aged 6–59 months were affected 2, 21. Despite ongoing national nutrition programs, these figures demonstrate the persistent burden of childhood anemia.

The prevalence of anemia varies across regions and between urban and rural areas. Studies from China, Equatorial Guinea, and Indonesia show higher rates in rural populations, often reflecting differences in socioeconomic conditions, maternal education, access to healthcare, dietary practices, sanitation, and environmental exposures [4–6, 25, 26]. In North Sumatera, Indonesia, for instance, 17.3% of rural children were anemic versus 12.7% of urban children [25]. These disparities underscore the importance of understanding local determinants of anemia to develop context-specific public health interventions.

Despite recommendations from the Indonesian Pediatric Society to routinely assess hemoglobin from age two and provide iron supplementation when indicated, systematic screening and interventions remain inconsistent, particularly in rural areas [21, 22, 27]. Barriers include limited healthcare infrastructure, low caregiver awareness, and insufficient integration into national programs [28, 29]. Therefore, while childhood anemia is highly prevalent in Indonesia, there is limited, region-specific, and up-to-date evidence on prevalence, urban–rural disparities, and associated risk factors in West Java. Addressing this gap is essential for designing targeted and effective public health strategies.

This study aimed to estimate the prevalence of childhood anemia and identify associated risk factors among children aged 6–59 months in urban and rural areas of West Java, Indonesia, with a focus on demographic, nutritional, and socioeconomic determinants. It also sought to examine urban–rural disparities in anemia prevalence and risk factors to inform evidence-based, context-specific public health strategies for anemia prevention and control in young children.

## Materials and methods

This study was a secondary cross-sectional analysis of data obtained from a seroepidemiological survey of hand, foot, and mouth disease (HFMD) conducted between November 2022 and January 2023. The original serosurvey aimed to assess antibody responses to HFMD-related viruses using serum samples. Venous blood was collected for antibody testing, and hemoglobin concentration was measured from whole blood samples during routine laboratory processing. Given the availability of hemoglobin data and the inclusion of apparently healthy children from both urban and rural settings using structured recruitment procedures, the dataset provided a suitable opportunity for secondary analysis to estimate the prevalence of anemia and examine associated risk factors within the same population.

The study included 562 apparently healthy children aged 6–59 months recruited from two primary healthcare centers: Garuda Primary Health Care in Bandung City (urban area) and Padalarang Primary Health Care in West Bandung Region (rural area), Indonesia. Bandung City is a densely populated urban area with a wide range of socioeconomic conditions and 45 primary healthcare facilities providing routine pediatric services. West Bandung Region is a predominantly rural area with lower population density and 38 primary healthcare centers serving dispersed communities. The two sites were selected to provide a representative sample of urban and rural populations in West Java and to capture potential regional differences in childhood anemia prevalence. Parents or legal guardians received a full explanation of the study procedures and provided written informed consent prior to participation. Children were excluded if blood collection was unsuccessful (e.g., sample clotting) or if they had severe illness requiring immediate medical treatment at the time of recruitment.

### Sampling method

**Fig. 1:**
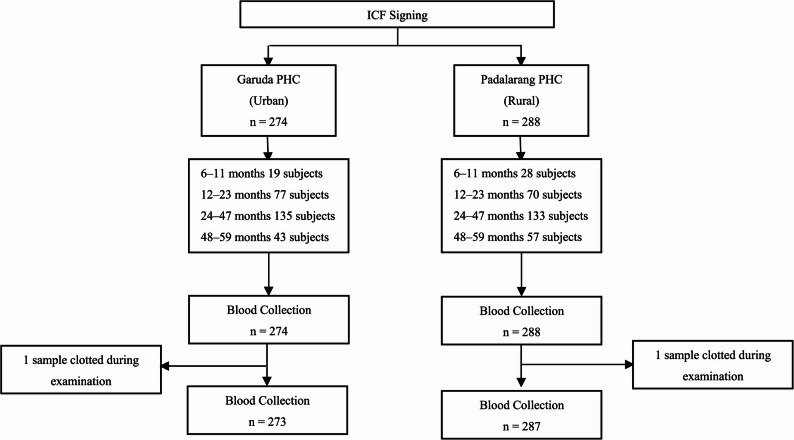
Flow chart of sample size selection

A total sampling approach was applied to all eligible participants enrolled in the original HFMD serosurvey. Recruitment at each site followed quota sampling based on age strata (6–11 months, 12–23 months, 24–47 months, and 48–59 months) to reflect the underlying age distribution of the population. Initially, 562 children were enrolled (274 from the urban site and 288 from the rural site). Two blood samples (one from each site) were excluded due to clotting during processing, resulting in 560 analyzable samples (Fig. 1).

### Blood collection and hemoglobin measurement

Venous blood samples (2–3 mL) were collected by trained healthcare personnel following standard aseptic procedures. Hemoglobin concentration was measured from whole blood using automated hematology analyzers (Sysmex XN-1000, Sysmex Corporation, Kobe, Japan) according to standard laboratory methods. Serum samples were separated by centrifugation at 3000 rpm for 10 min at room temperature, aliquoted, and stored at − 80 °C for HFMD antibody testing. The anemia assessment in this study was based solely on hemoglobin concentration.

### Definition of variables

Anemia was defined according to World Health Organization (WHO) criteria as a hemoglobin (Hb) concentration < 11.0 g/dL in children aged 6–59 months. The severity of anemia was classified as mild (10.0–10.9 g/dL), moderate (7.0–9.9 g/dL), and severe (< 7.0 g/dL) [8].

Child characteristics included immunization and nutritional status. Immunization status was categorized as complete or incomplete based on the child’s vaccination record. Nutritional status was assessed using anthropometric measurements, including length/height and weight, and interpreted according to the WHO Child Growth Standards (2006). Height-for-age z-score (HAZ) < − 2 standard deviations (SD) was defined as stunting, weight-for-age z-score (WAZ) < − 2 SD as underweight, and weight-for-height z-score (WHZ) < − 2 SD as wasting. Overweight or obesity was defined as WHZ > + 2 SD. Length was measured in children under 2 years using an infantometer, while standing height was measured in children aged 2 years or older using a calibrated stadiometer [10]. All measurements were performed by trained personnel following standardized procedures.

Parental characteristics included household income and education level. Parental income was categorized as above or below the regional minimum wage. Fathers’ and mothers’ education levels were classified as primary school or below, junior high school, senior high school, or college and above.

### Statistical analysis

Data were analyzed using SPSS version 26 (IBM Corp., Armonk, NY, USA). Categorical variables were compared using the chi-square test. Variables considered clinically or biologically relevant based on prior literature were retained in the multivariable model regardless of bivariate significance to control for potential confounding. Adjusted odds ratios (AORs) with 95% confidence intervals were calculated. Statistical significance was defined as *p* < 0.05.

## Results

A total of 560 children aged 6–59 months were included in the analysis, with 274 children from the urban site and 286 from the rural site. Overall, 131 children (23.4%) were anemic, with 70 children (25.6%) in urban areas and 61 children (21.3%) in rural areas affected. The sample was balanced by sex, with 70 males (23.3%) and 61 females (23.6%) classified as anemic. Among age groups, 62 children aged 6–23 months (32.0%) and 69 children aged 24–59 months (18.9%) were anemic. Regarding nutritional status, stunting was present in 36 children (31.0%) who were anemic. Other indicators, including underweight, wasting, and overweight/obesity, were also recorded and summarized. Household characteristics showed that 87 children (27.6%) from families with income below the regional minimum wage were anemic, compared with 44 children (18.0%) from families above this threshold. Parental educational levels and immunization status were collected and described. These descriptive data provide an overview of the population characteristics and anemia prevalence before examining associations with potential risk factors Table 1.

**Table 1:** Characteristic of anemia among children 6–59 months (*n* = 560)

Characteristics	Prevalence		*P* value*	OR (95% CI)
	Anemia (*n* = 131)	Non-Anemia (*n* = 429)		
Location			0.220	
Urban	70 (25.6%)	203 (74.4%)		1.21 (0.89–1.63)
Rural	61 (21.3%)	226 (78.7%)		1.0
Age of Children			*<0.001	
6–23 months	62 (32.0%)	132 (68.0%)		1.70 (1.23–2.28)
24–59 months	69 (18.9%)	297 (81.1%)		1.0
Sex of child			0.934	
Male	70 (23.3%)	231 (76.7%)		1.0
female	61 (23.6%)	198 (76.4%)		1.01 (0.75–1.37)
Parent’s income			*0.007	
Above regional minimum wage	44 (18.0%)	201 (82.0%)		1.0
Below regional minimum wage	87 (27.6%)	228 (72.4%)		1.54 (1.11–2.12)
Father’s educational status			0.208	
≤ Primary School	6 (15.0%)	34 (85.0%)		0.90 (0.37–2.19)
Junior High School	34 (26.2%)	96 (73.8%)		1.36 (0.76–2.42)
Senior High School	78 (25.0%)	234 (75.0%)		1.50 (0.88–2.55)
≥ College	13 (16.7%)	65 (83.3%)		1.0
Mother’s educational status			0.389	
≤ Primary School	14 (26.9%)	38 (73.1%)		1.61 (0.84–3.11)
Junior High School	34 (22.7%)	116 (77.3%)		1.30 (0.78–2.39)
Senior High School	69 (25.2%)	205 (74.8%)		1.51 (0.90–2.54)
≥ College	14 (16.7%)	70 (83.3%)		1.0
Immunization Status			0.870	
Complete Immunization	71 (23.1%)	236 (76.9%)		1.0
Incomplete Immunization	60 (23.7%)	193 (76.3%)		1.02 (0.76–1.38)
HAZ			*0.029	
Stunting	36 (31.0%)	80 (69.0%)		1.45 (1.05–2.01)
Normal	95 (21.4%)	349 (78.6%)		1.0
WAZ			0.645	
Underweight	18 (21.4%)	66 (78.6%)		0.90 (0.58–1.40)
Normal	113 (23.7%)	363 (76.3%)		1.0
WHZ			0.159	
Wasting	12 (18.5%)	53 (81.5%)		0.74 (0.43–1.26)
Overweight/Obesity	6 (14.0%)	37 (86.0%)		0.56 (0.26–1.19)
Normal	113 (25.0%)	339 (75.0%)		1.0

*) Chi-square test

These statistics showed that 25.6% in urban areas and 21.3% in rural areas were anemia. The variables that were statistically associated with anemia in the urban area were: the child’s age (*p* < 0.001), children with parents’ income below the regional minimum wage (*p* = 0.007), and stunting children (*p* = 0.029). In rural areas, no variables had a significant relationship with anemia.

**Fig. 2:**
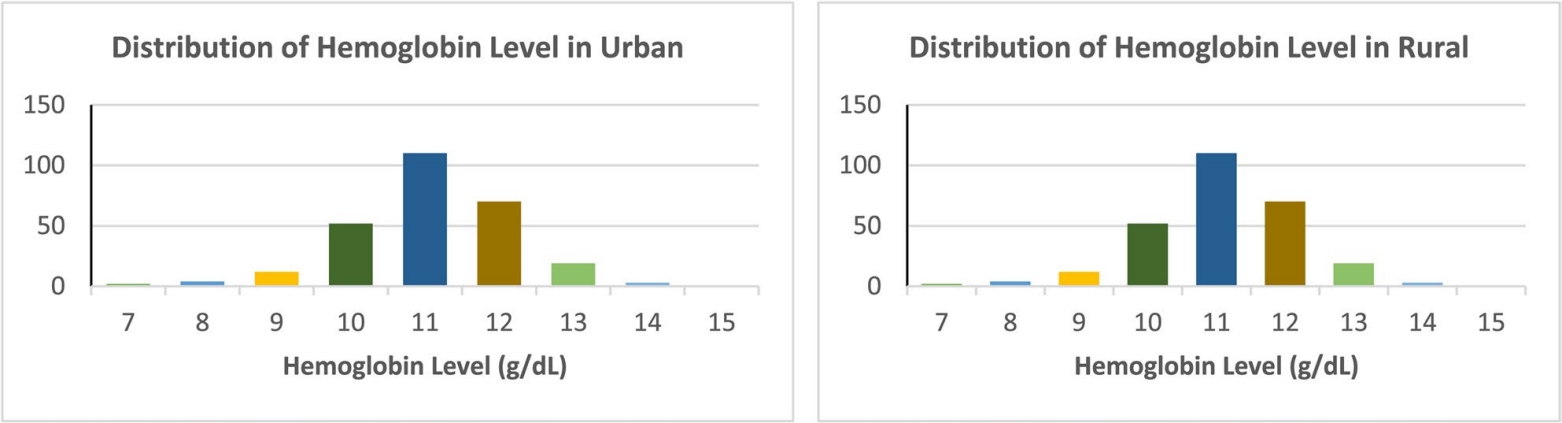
Distribution of hemoglobin level among urban and rural

The distribution of hemoglobin levels (g/dL) is presented in Fig. 2. A child was considered to be anemic when the estimated hemoglobin level was < 11.0 g/dL. Children were classified as severe anemia (< 7.0 g/dL), moderate anemia (7.0–9.9 g/dL), and mild anemia (10.0–10.9 g/dL). The severity of anemia is presented in Fig. 2.

The results from logistic regression analysis presented in Table 2 showed that the model for any level of anemia was statistically significant. The results showed that children aged 6–23 months (Adjusted Odds Ratio (AOR) = 2.17; 95% CI: 1.44–3.26; *p* < 0.001), and aged 24–59 months (AOR = 1; 95% CI: *p* < 0.001). Stunting children (AOR = 1.71; 95% CI: 1.07–2.72; *p* = 0.025) and children with parents income below regional minimum wage (AOR = 1.73; 95% CI: 1.14–2.63; *p* = 0.010) were more likely to be anemic.

**Table 2:** Determinants of any level of anemia among children 6–59 months using multiple logistic regression

Characteristics	Adjusted Odds Ratio (95% CI)	*P* Value
Age of children (in months)		
6–23 month	2.17 (1.44–3.26)	**< 0.001**
24–59 month	1	
Stunting		
Yes	1.71 (1.07–2.72)	**0.025**
No	1	
Parent’s income		
Above regional minimum wage	1	
Below regional minimum wage	1.73 (1.14–2.63)	**0.010**

## Discussion

This study found that childhood anemia remains a substantial public health issue in West Java, Indonesia, with prevalence of 25.6% in urban areas and 21.3% in rural areas. Although prevalence was slightly higher in urban children, the difference was not statistically significant, indicating a comparable burden across both settings. Among nutritional parameters, stunting was the only factor significantly associated with anemia, with stunted children 1.71 times more likely to be anemic [14, 30–33]. Age and socioeconomic status were also significant determinants, with children aged 6–23 months and those from lower-income households showing higher anemia risk [14, 34, 35]. Immunization status, underweight, wasting, and overweight/obesity were not significantly associated with anemia [15, 25, 36, 37].

The slightly higher prevalence observed in urban areas aligns with findings by Gang Gao et al. [12], but contrasts with studies in Equatorial Guinea and Peru, which reported higher anemia prevalence in rural populations [5, 6]. In North Sumatera, Indonesia, rural children were more affected [25]. Age-related anemia risk and the association with stunting are consistent with evidence from Bangladesh, China, and Haiti [14, 16, 17, 34, 35]. Socioeconomic status as a determinant is also well-documented across low- and middle-income countries [15, 25, 36, 37], with poverty influencing dietary quality, healthcare access, and overall child development [38–43].

The absence of statistically significant urban–rural differences may reflect contextual and environmental factors rather than a true lack of disparity. Urban households may have higher incomes but often rely on processed or convenience foods low in iron and other micronutrients [13, 23]. Rural households, while consuming more locally sourced foods, may face limited healthcare access and fewer fortified foods [44, 45]. Stunting reflects chronic undernutrition and cumulative exposure to infections, making it a stronger predictor of anemia than acute indicators such as underweight or wasting [32, 33, 46–48]. The higher risk among younger children is biologically plausible, as rapid growth and increased iron requirements during the first two years of life elevate susceptibility to iron deficiency [49, 50]. Iron deficiency accounts for nearly half of anemia cases globally, though infections and deficiencies of other micronutrients also contribute [5, 8].

These findings highlight the need for context-sensitive interventions targeting both urban and rural populations. Strategies should include iron supplementation, promotion of iron-rich and fortified foods, routine screening at primary healthcare centers and Posyandu, and caregiver education [9, 21, 51–53]. Existing best practices in Central Java and East Nusa Tenggara, combining supplementation, fortification, and community education, demonstrate measurable reductions in childhood anemia and could be adapted for other regions [34, 54, 55]. Integrating stunting prevention alongside anemia interventions is critical, as these conditions share underlying nutritional and socioeconomic determinants. Early intervention during the first 1000 days of life is particularly important to prevent chronic anemia and its long-term developmental consequences [34, 54, 55].

This study benefits from a well-structured dataset including both urban and rural populations, enabling direct comparison within a consistent population. Age-stratified quota sampling and standardized anthropometric and hemoglobin measurements enhance the reliability of prevalence estimates. The study also examined multiple potential risk factors, including demographic, nutritional, and socioeconomic variables, offering a comprehensive assessment of childhood anemia determinants.

Several limitations should be acknowledged. First, the study did not assess early feeding practices, complementary feeding, dietary intake, infectious status, iron supplementation, or maternal gestational conditions, which may influence anemia risk. Second, anemia classification relied solely on hemoglobin concentration, without additional hematological parameters, limiting differentiation of anemia types. Third, the selection of urban and rural sites with similar cultural and socioeconomic characteristics may have reduced detectable differences, complicating urban–rural comparisons. Finally, while the sample size was sufficient for prevalence estimation, generalizability to other regions of Indonesia with different environmental and social contexts may be limited.

Despite these limitations, the study provides important insights for national anemia prevention programs, emphasizing the need for comprehensive interventions addressing nutrition, socioeconomic disparities, and early childhood health to reduce anemia prevalence and improve child outcomes in both urban and rural settings.

## Conclusion

In the current study showed that children both in rural areas and urban areas, can have anemia. Children aged 6–23 months, stunting children, and children with parents’ income below regional minimum wage were identified as factors associated with anemia in children. Further research and evaluation is needed in detection and monitoring of risk factors through a multisectoral approach. Therefore, it is important to know the etiological pattern of anemia in society so that we can take more effective actions and strategies at preventing anemia in children.

## Data Availability

The data supporting the findings of this study are available from the corresponding author upon reasonable request.
